# Transhepatic Transcatheter Pulmonary Valve Replacement

**DOI:** 10.1016/j.jaccas.2024.102475

**Published:** 2024-08-21

**Authors:** Michael R. Hainstock, Barbara A. Castro, Stephanie R. Kidney, D. Scott Lim

**Affiliations:** a Division of Pediatric Cardiology, Department of Pediatrics, University of Virginia, Charlottesville, Virginia, USA; b Division of Pediatric Cardiac Anesthesia, Department of Anesthesia, University of Virginia, Charlottesville, Virginia, USA; c Division of Cardiology, Department of Medicine, Division of Cardiology, University of Virginia, Charlottesville, Virginia, USA

**Keywords:** congenital heart defect, pulmonic valve, right-sided catheterization, valve replacement

## Abstract

Transcatheter pulmonary valve replacement (TPVR) is complicated in patients without adequate femoral or internal jugular vascular access. Transhepatic vascular access has been shown to be safe and effective across a spectrum of diagnostic and interventional procedures. Closure of the hepatic venous tract can be accomplished with a multitude of readily available vascular occlusion devices. The rates of major adverse events are low: 5% to 8% with hemoperitoneum and complete heart block are most significant. To our knowledge, this is the first report of using transhepatic access for TPVR; closure of the hepatic venous tract was achieved with an Amplatzer vascular plug type II.

## Graphical Abstract

**Figure:**
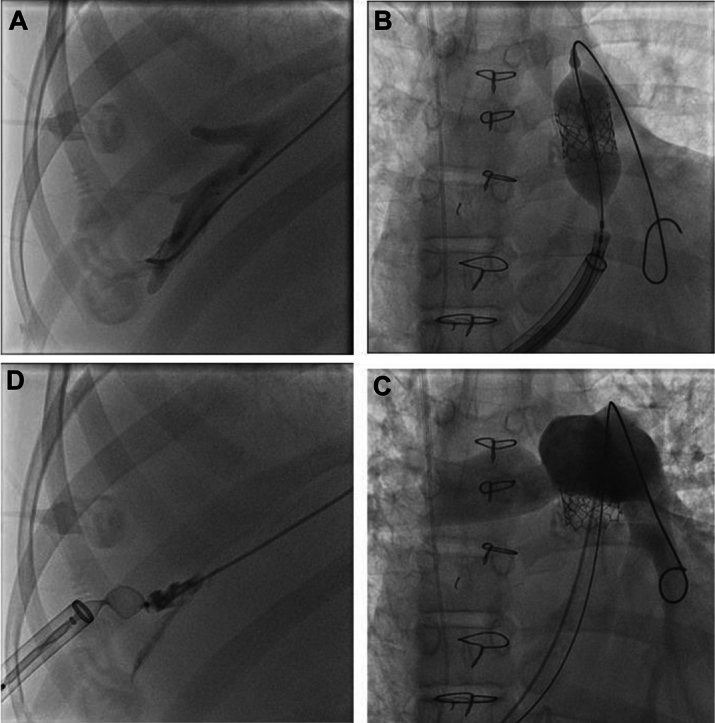

A 16-year-old adolescent boy was referred to our congenital cardiac interventional team with echocardiographic findings of combined pulmonary homograft stenosis (peak 67 mm Hg/mean 42 mm Hg) and severe regurgitation. The results of his physical exam included weight 67 kg, height 170 cm, hemoglobin 14.9 g/dL, oxygen saturation 100%, and a grade 5 systolic ejection murmur at the left upper sternal border. Owing to bilateral femoral vein occlusions, transcatheter intervention was attempted via ab internal jugular vein approach but was unsuccessful despite multiple manipulative techniques, including the use of progressively more rigid wires (Lunderquist/Myer) along with alternating the right pulmonary artery and left pulmonary artery wire positions. Indeed, even high-pressure angioplasty balloons were unable to be advanced by 2 experienced operators for preparation of the landing zone; the wire/balloon complex repeatedly prolapsed into the right ventricle apex. Although subclavian venous access was an option, the operators did not believe that approach would confer a significant advantage over the internal jugular vein; therefore, the case was aborted.

**Learning Objectives**

• To view transhepatic access as an important tool for transcatheter diagnostic and interventional procedures including the use of large (>20-F) vascular sheaths.
• To understand the potential complications (heart block, peritoneal hemorrhage) associated with transhepatic access as being important for the intraprocedural and postprocedural use and management of resources.

## Past Medical History

The patient had an original diagnosis of left-sided outflow tract obstructive lesions and had undergone a balloon aortic valvuloplasty followed by a Ross procedure as an infant. Subsequently, his pulmonary valve was surgically replaced with a 21-mm pulmonary homograft when he was 3 years old.

## Differential Diagnosis

The differential diagnosis of right ventricular hypertension included right ventricular outflow tract obstruction, pulmonary artery stenosis, pulmonary hypertension, chronic lung disease, chronic thromboembolic disease, left-sided inflow obstruction, and left ventricular diastolic dysfunction

## Investigations

In a multidisciplinary discussion, a perventricular hybrid approach was presented but declined by the surgical team, who preferred an open surgical pulmonary valve replacement. The family was counseled on the options of a surgical pulmonary valve replacement via a third-time redo sternotomy versus attempted transhepatic transcatheter pulmonary valve replacement (TPVR). Their preference was the transcatheter approach because his previous interventions had been complicated by bacteremia in addition to his mild pectus deformity (Haller index 3.0).

## Management

**Figure 1:**
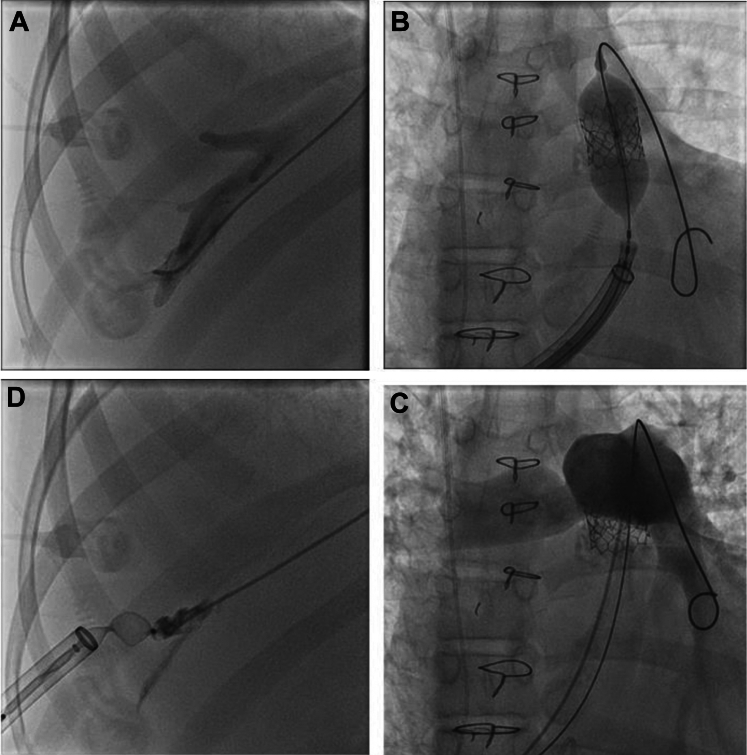
Transhepatic Access and Pulmonary Valve Delivery (A) Injection of contrast material in the hepatic vein via end-hole catheter from the right internal jugular vein used for target-directed transhepatic access. (B) Delivery of the 23-mm Sapien S3 Ultra via 26-F Gore DrySeal long sheath (end-hole catheter from internal jugular used for target-guided hepatic access). (C) Angiograph after valve deployment demonstrating no pulmonary insufficiency (contrast material delivered via angiographic catheter advanced from hepatic access). (D) Injection of contrast material in the hepatic vein with partial deployment of the 16-mm Amplatzer vascular plug type II demonstrating no extrahepatic extravasation of contrast material.

**Figure 2:**
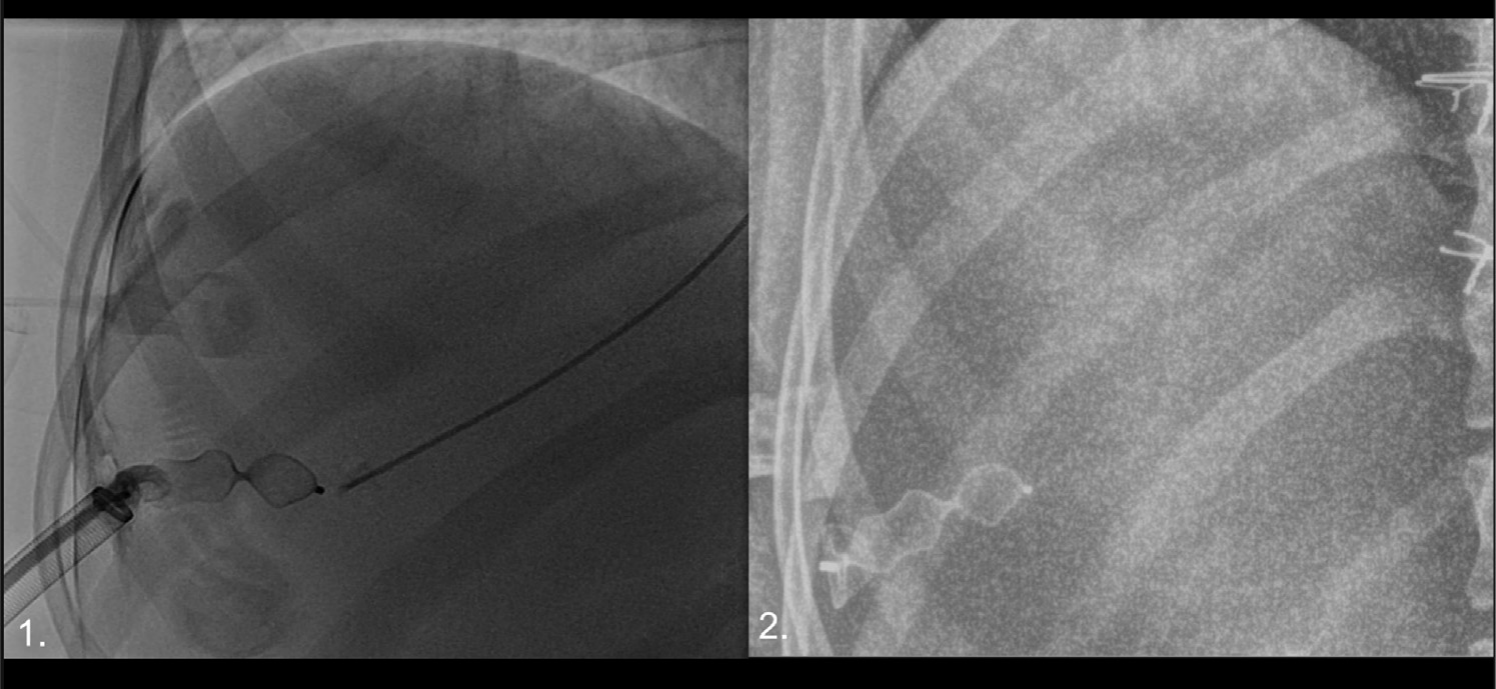
Occlusion of the Hepatic Venous Tract (Left) Fluoroscopic view demonstrating fully deployed 16-mm Amplatzer vascular plug type II. (Right) Abdominal radiograph the morning after the procedure demonstrating fully formed and stable position of the hepatic occlusion device.

At the index procedure, internal jugular vein access was obtained and retrograde hepatic venography performed to guide transhepatic access (Figure 1). After percutaneous modified Seldinger access into the hepatic vein, a distal pulmonary artery wire position was achieved using a double-curved Lunderquist wire. The hepatic access tract was serially dilated, and ultimately a 26-F sheath (Gore DrySeal) was advanced across the homograft, where high-pressure balloon angioplasty was performed with 16-mm, 18-mm, and 20-mm balloons (Atlas Gold, Bard/Beckinson Dickenson). A 23-mm Sapien Ultra transcatheter heart valve (Edwards Lifesciences) was then delivered in the pulmonary position. Subsequent hemodynamics demonstrated a reduction in right ventricular systolic pressure to 40 mm Hg (baseline 70 mm Hg) with a <5-mm Hg residual gradient (baseline gradient 40 mm Hg), with no pulmonary insufficiency by angiography. A 16-mm Amplatzer vascular plug type II (Abbott) was used to occlude the hepatic venous tract (Figure 2), and angiography from the internal jugular vein access demonstrated no vascular leak.

## Outcome and Follow-Up

The patient was observed overnight. with no change in his hemoglobin 4 hours after the procedure. An echocardiogram the next morning showed excellent valve function with mild stenosis and no regurgitation. He was discharged the morning after the procedure with no complications noted. At the 18-month follow-up visit, the Sapien S3 Ultra transcatheter heart valve had excellent function with mild stenosis and no regurgitation

## Discussion

TPVR has been widely used as an alternative treatment to surgery. Vascular access is commonly performed using the femoral or internal jugular vein, with demonstrated success. Additionally, perventricular access in small children, or those with inadequate alternative vascular access, has proved to be safe and effective.^1^^,^^2^

Vascular access through a transhepatic approach has also demonstrated its utility as an alternative site for both diagnostic and interventional cases.^3^^,^^4^ Other reports have shown the potential for intervention in lesions such as secundum atrial septal defect (ASD) closure, pulmonary valve dilation, pulmonary artery dilation,^5^ MitraClip,^6^ left atrial appendage occlusion,^7^ and sinus venosus ASD closure.^8^ The use of large sheath sizes has also been demonstrated with a 24-F used for MitraClip and a 14-F used for sinus venosus ASD closure.

Closure of the hepatic tract has been performed using a variety of occlusion devices. These options include use of Gelfoam, coils, vascular plugs,^5^ or a hemostatic sponge.^9^ In patients with central venous lines are placed via a transhepatic approach, removal without using an occlusion device has also been shown to be a reasonable strategy.

Adverse events occur infrequently; the incidence of serious adverse events is approximately 5%.^5^^,^^10^^,^^11^ Qureshi et al^11^ published the largest single-center review of >120 procedures performed through transhepatic access, with significant adverse events occurring in 8%. These events included hemoperitoneum (1 requiring laparotomy), hemothorax, and complete heart block necessitating placement of a pacemaker in 4 patients, of whom 2 subsequently experienced resolution. Additionally, the French size of the sheath was not associated with adverse events. The operator should be aware of these potential complications in addition to retroperitoneal bleeding, pneumothorax, and infection.

Our patient also has shown transhepatic access as a viable alternative for TPVR using otherwise standard techniques, with no significant adverse event.

## Conclusions

Transhepatic access for transcatheter pulmonary valve replacement is a viable, though not low-risk, alternative vascular option when other more traditional routes are not available. Closure of the venous access tract can be performed with standard vascular occlusion devices.

## Funding Support and Author Disclosures

The authors have reported that they have no relationships relevant to the contents of this paper to disclose.

## References

[1] Cubeddu R.J., Hijazi Z.M. (2011). Bailout perventricular pulmonary valve implantation following failed percutaneous attempt using the Edwards Sapien transcatheter heart valve. Catheter Cardiovasc Interv.

[2] Ng L.Y., Al-Alawi K., Breatnach C. (2021). Hybrid subxiphoid perventricular approach as an alternative access in neonates and small children undergoing complex congenital heart interventions. Pediatr Cardiol.

[3] Shim D., Lloyd T.R., Cho K.J., Moorehead C.P., Beekman R.H. (1995). Transhepatic cardiac catheterization in children: evaluation of efficacy and safety. Circulation.

[4] Singh S.M., Neuzil P., Skoka J. (2011). Percutaneous transhepatic venous access for catheter ablation procedures in patients with interruption of the inferior vena cava. Circ Arrhythm Electrophysiol.

[5] Ebeid M.R. (2007). Transhepatic vascular access for diagnostic and interventional procedures: techniques, outcome, and complications. Catheter Cardiovasc Interv.

[6] de Brito F.S., Nasser F., Gobbo R. (2018). Percutaneous transhepatic mitral valve repair with the MitraClip system. JACC Cardiovasc Interv.

[7] Huang H.D., Murphy J.J., Sharma A., Kavinsky C.J., Poulin M.F. (2019). Novel transhepatic percutaneous approach for left atrial appendage occlusion using a watchman device. JACC Cardiovasc Interv.

[8] Alkhouli M., Campsey D.M., Higgins L., Badhwar V., Diab A., Sengupta P.P. (2018). Transcatheter closure of a sinus venosus atrial septal defect via transhepatic access. JACC Cardiovasc Interv.

[9] Haddad R.N., Maleux G., Bonnet D., Malekzadeh-Milani S. (2020). Transhepatic atrial septal defect closure: simple way to achieve haemostasis in a patient with important co-morbidities. Cardiol Young.

[10] Erenberg F.G., Shim D., Beekman R.H. (1998). Intraperitoneal hemorrhage associated with transhepatic cardiac catheterization: a report of two cases. Cathet Cardiovasc Diagn.

[11] Qureshi A.M., Prieto L.R., Bradley-Skelton S., Latson L.A. (2014). Complications related to transhepatic venous access in the catheterization laboratory: a single center 12-year experience of 124 procedures. Catheter Cardiovasc Interv.

